# A nonenzymatic electrochemical glucose sensor depending on a NiNPs/Co_3_O_4_/functionalized graphene oxide-modified working electrode

**DOI:** 10.55730/1300-0527.3801

**Published:** 2026-05-04

**Authors:** Sümeyra PEKMEZ, Muhammet GÜLER

**Affiliations:** Department of Chemistry, Faculty of Science, Van Yüzüncü Yıl University, Van, Turkiye

**Keywords:** Glucose, functionalized graphene oxide, NiNPs@Co_3_O_4_, sensor

## Abstract

In the present research, the aim was to develop a novel electrochemical enzyme-free glucose sensor dependent upon a nickel nanoparticles (NiNPs)/cobalt (II, III) oxide (Co_3_O_4_)/carboxylated graphene oxide (GO-COOH)-modified glassy carbon (GC) electrode. The electrocatalytic performance of the electrode was tested using various electrochemical methods such as amperometry and cyclic voltammetry (CV). The NiNPs@Co_3_O_4_/GO-COOH/GC electrode showed noticeably superior electrocatalytic behavior for glucose oxidation in 0.1 M NaOH media, ascribed to the good synergistic impact between NiNPs, Co_3_O_4_, and GO-COOH. The amperometric response of the NiNPs@Co_3_O_4_/GO-COOH/GC electrode to glucose exhibited two linear determination ranges at +0.55 V potential. The first one was between 4.8 × 10^−7^ and 9.5 × 10^−4^ M and the second one was between 9.5 × 10^−4^ and 4.87 × 10^−3^ M. The limit of detection was 1.4 × 10^−7^ M dependent upon a signal-to-noise ratio of 3. The sensor displayed good performance factors such as selectivity, reproducibility, and stability. Furthermore, the suitability of the sensor for detecting glucose in biological fluids proved satisfactory.

## 1. Introduction

Glucose is, without a doubt, a compound highly critical for life. The glucose concentration in body fluids is considered an indicator of a properly functioning organism. Blood glucose levels are an important indicator in many diseases, including diabetes, obesity, and cancer [1]. In human blood, glucose concentrations that are too low can cause cell death, while those that are too high can lead to a cessation of cellular activity [2]. When blood glucose concentrations are high, glucose irreversibly reacts with the amino groups of amino acids to form glycosylated proteins, which are important biomarkers for atherosclerosis, cancer, and Alzheimer’s disease [3]. The literature contains research that refers to Alzheimer’s disease as “type 3 diabetes” and suggests that many unexplained Alzheimer’s features, such as cell death and plaque formation in the brain, are directly linked to disruptions in insulin signaling [4]. In type 1 diabetes, the body does not produce insulin, requiring periodic insulin injections, and this type is more common in young people [5]. In type 2 diabetes, the body cannot effectively use the insulin it produces, and it typically begins in adulthood [6]. In developing or developed countries where access to food is very easy, type 2 diabetes is considered an epidemic [7]. There is no cure for diabetes with any technology and more than 1 million people die each year due to poorly controlled blood glucose. Therefore, accurate detection and evaluation of blood glucose levels are of paramount importance [8].

The International Diabetes Federation describes diabetes as a global burden [9]. In 1997, the cost of diabetes in the US was 98 billion, while in 2002, in just 5 years, it had increased by 35% to 132 billion. This amount represents 20% of total personal healthcare spending [10]. Studies have shown that the treatment costs for diabetic patients who have developed complications related to diabetes are more than double the treatment costs for patients without complications. High levels of glycated protein are known to cause serious health problems such as kidney failure, heart attacks, permanent retinal damage, limb amputation, permanent paralysis, depression, liver disorders, gum disease, hearing loss, high blood pressure, and low-density lipoprotein cholesterol [11]. Therefore, continuous efforts are being made for the early diagnosis of diabetes to prevent its progression and the onset of complications, with the aim of preventing it from becoming a global burden [12]. Frequent and long-term monitoring of blood glucose concentration and maintaining it within the normal range is important for preventing complications and improving survival rates [13].

Biosensors are a critical part of modern life, and electrochemical sensors occupy a significant place in both the scientific and patent literature because they are reliable for working with electrical parameters; provide high sensitivity, stability, simplicity, and cost-effectiveness; and are implantable, portable, fast, and easy to process data and miniaturize [14]. Electrochemical-based systems are primarily used for glucose sensors [15]. Sensors that use enzymes as molecular recognition tools have advantages such as high selectivity and sensitivity. However, the enzyme’s instability, the difficulty and high cost of enzyme immobilization on the surface, its low reproducibility, and its sensitivity to pH, humidity, and temperature changes have led researchers to investigate glucose sensors that do not require the use of enzymes. Nonenzymatic electrochemical sensors, based on the direct electrocatalytic oxidation of glucose on the electrode, have advantages such as high sensitivity, long-term stability, low cost, and easy production, eliminating deviation and increasing reliability [16,17].

Glassy carbon electrodes (GCEs) are widely used as the basic electrode in the production of nanostructured electrodes due to their cost-effectiveness, mechanically stable compact solid structure, impermeability to gases and liquids, high porosity, wide potential range, and ease of surface modification. The electrochemical properties of GCEs can be easily altered through modification. GCEs have been frequently used in glucose sensors in the literature [18].

The incorporation of nanomaterials into electrochemical sensors offers advantages such as reducing the overpotential of electrochemical reactions, making irreversible reactions reversible in unmodified electrodes, and providing multiple detection capabilities. Due to their large specific surface area and very good biocompatibility, many nanomaterials such as Au nanoparticles, graphene, carbon nanotubes, and their composites have been used in electrochemical applications, and these materials have been observed to exhibit tunneling behavior from the electrode to the active site [19]. Functionalization of graphene with metal oxide nanoparticles increases its sensitivity to glucose [20]. Metal oxide nanoparticles are effective catalysts due to their high proportion of free-valence surface atoms and can provide electrochemical reversal for redox reactions [21].

To date, Ni- and/or Co_3_O_4_-based working electrodes have been reported for the nonenzymatic determination of glucose. Among these studies, the most noteworthy are those supported by graphene oxide. Graphene oxide contains oxygen-containing functional groups such as hydroxyl, carboxyl, and epoxy. Due to the presence of these functional groups, graphene oxide can be modified with metals/metal oxides and various functional groups. It has been reported that modifying the active surface area of the working electrode with oxygen-containing functional groups increases both the active surface area and the sensitivity of the working electrode. Furthermore, nickel has shown excellent performance for nonenzymatic glucose determination due to its exceptional electrochemical properties. In addition, Co_3_O_4_ has been reported to be an ideal catalyst for glucose determination due to its Co^2+^/Co^3+^ redox pair [22–26]. Therefore, in our study, graphene oxide was synthesized using the improved Hummers method, and then GO-COOH was obtained with chloroacetic acid. Subsequently, Ni@Co_3_O_4_ composite was deposited on the GO-COOH surface.

In the present research work, an electrochemical nonenzymatic glucose sensor was constructed depending upon a NiNPs/Co_3_O_4_/GO-COOH modified glassy carbon (GC) electrode. The synthesized NiNPs/Co_3_O_4_/GO-COOH nanocomposite was evaluated by various techniques such as X-ray photoelectron spectroscopy (XPS), X-ray diffraction (XRD), and scanning electron microscopy (SEM). Modification of the GC electrode was achieved using the drop-casting method. The prepared NiNPs/Co_3_O_4_/GO-COOH/GC electrochemical sensor was investigated for sensitivity, selectivity, linearity, limit of detection (LOD), limit of quantification (LOQ), reusability, reproducibility, and storage stability. Further, the applicability of the sensor was tested in serum samples. A commercial glucometer was used to compare the results.

## 2. Materials and methods

### 2.1. Chemicals and reagents

Graphite (Gr, powder, <20 μm, synthetic), cobalt(II) nitrate hexahydrate (Co(NO_3_)_2_.6H_2_O, ≥98), nickel(II) chloride hexahydrate (NiCl_2_.6H_2_O, 99.9%), dopamine (DA) HCl (≥98%), glucose (Glu, ≥99.5%), ascorbic acid (AA, reagent grade), L-tryptophan (≥98%), potassium permanganate (KMnO_4_, ≥98%), phosphoric acid (H_3_PO_4_, 85%), D-(−)-sucrose (≥99%), sulfuric acid (H_2_SO_4_, 98%), urea (reagent grade), hydrochloric acid (HCl, 37%), uric acid (UA, ≥99%), potassium chloride (KCl, ≥99%), and sodium hydroxide (NaOH, ≥98%) were obtained from Sigma-Aldrich. Hydrazine hydrate (NH_2_NH_2_.H_2_O) was procured from Thermo Scientific. Ethanol absolute (C_2_H_5_OH), hydrogen peroxide (H_2_O_2_, ≥35%), chloroacetic acid (ClCH_2_COOH, for synthesis), and nitric acid (HNO_3_, ≥65%) with every other chemical were provided by Merck.

### 2.2. Measurements

Autolab PGSTAT128N with FRA 32 M was used for the electrochemical experiments. The electrochemical system consisted of a three-electrode system: a working electrode, a counter electrode, and a reference electrode. The working, counter, and reference electrodes were GC and modified GC, platinum (Pt) wire, and silver/silver chloride (Ag/AgCl) electrodes, respectively. The structure of the prepared f-GO-COOH, Co_3_O_4_, Co_3_O_4_/GO-COOH, and NiNPs@Co_3_O_4_/GO-COOH were investigated using SEM (Sigma 300, Zeiss), XRD (Panalytical), and XPS (SPECS Surface Nano Analysis GmbH, Germany).

### 2.3. Synthesis of graphene oxide and functionalized graphene oxide

Here graphene oxide (GO) was prepared via the modified Hummers method [27]. To obtain carboxylated graphene oxide (GO-COOH), 0.5 g of GO was put into a flask containing 100 mL of bidistilled water and stirred (1 h) and then sonicated (1 h). Next, 2.5 g of KOH and 5 g of ClCH_2_COOH were added to the mixture. The mixture was stirred for 20 h at 95 ± 2 °C in an oil bath. Following thorough filtering and cleaning with double distilled water (5 × 30 mL) and then ethanol (2 × 30 mL), the synthesized GO-COOH was vacuum dried at 60 °C [28,29].

### 2.4. Preparation of Co_3_O_4_

In order to synthesize Co_3_O_4_, 0.6 g of Co(NO_3_)_2_.6H_2_O and 0.9 g of urea were put into a beaker containing 60 mL of ultrapure water. The homogeneous mixture was stirred for about 30 min and then transferred to a 100-mL Teflon-lined stainless steel autoclave at 160 °C for 10 h [30]. The material was cooled to laboratory temperature, washed with water and ethanol, and vacuum-dried at 60 °C. The powder was heated at in a muffle furnace at 550 °C for 3 h in air atmosphere. The compound obtained was Co_3_O_4_.

### 2.5. Preparation of Co_3_O_4_/GO-COOH and NiNPs@Co_3_O_4_/GO-COOH

To synthesize Co_3_O_4_/GO-COOH, 20 mg of GO-COOH, 60 mg of Co_3_O_4_, and 30 mL of ethylene glycol were put into 50-mL beaker, in that order. The mixture was dispersed homogeneously sequentially for 1 h each using a magnetic stirrer and an ultrasonic bath. After that, the mixture was stirred magnetically for 12 h at laboratory temperature. After the mixture was filtered and successively cleaned with double-distilled water and absolute ethanol, it was allowed to dry at 60 °C in a vacuum oven [31].

To prepare NiNPs@Co_3_O_4_/GO-COOH, 50 mg of Co_3_O_4_/GO-COOH and 30 mL of ethylene glycol were added to a 50-mL beaker. The mixture was dispersed for 1 h using an ultrasonic bath. The mixture was then stirred for 3 h at 60 °C in an oil bath after 0.25 mL of 1 M NiCl_2_.6H_2_O (0.0594 g), 1.25 mL of NH_2_NH_2_.H_2_O, and 3 mL of 1.0 M NaOH were added to the mixture. Finally, the mixture was cleaned using ultrapure water and absolute ethanol by means of a centrifuge at 8000 rpm for 10 min and vacuum-dried at 60 °C [32].

### 2.6. Preparation of working electrodes

In order to modify the bare GCE, firstly the electrode was polished using Al_2_O_3_ slurry and then cleaned in absolute ethanol and then HNO_3_/H_2_O (1:1) for 3 min by means of an ultrasonic bath and dried at room temperature. Next, 1 mg of NiNPs@Co_3_O_4_/GO-COOH and 1 mL of bidistilled water were put into a test tube and dispersed homogeneously for 20 min using the ultrasonic bath. Then 6 μL of NiNPs@Co_3_O_4_/GO-COOH dispersion was cast on the cleaned GC electrode and it was vacuum-dried at 50 °C. Co_3_O_4_/GC, GO-COOH/GC, and Co_3_O_4_/GO-COOH/GC electrodes were prepared using the same method.

## 3. Results and discussion

### 3.1. Characterization of materials

**Figure 1:**
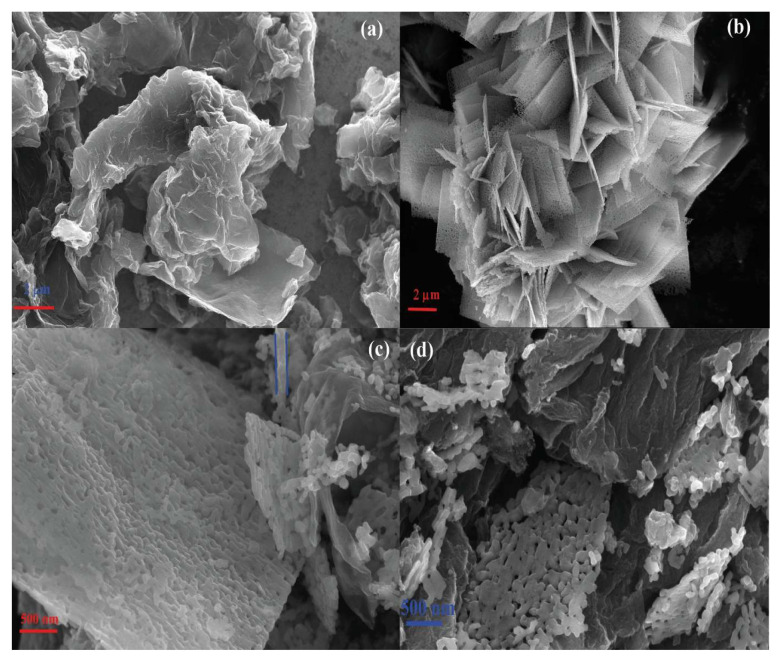
SEM images of GO-COOH (a), Co_3_O_4_ (b), Co_3_O_4_/GO-COOH (c), and NiNPs@Co_3_O_4_/GO-COOH (d).

**Figure 2:**
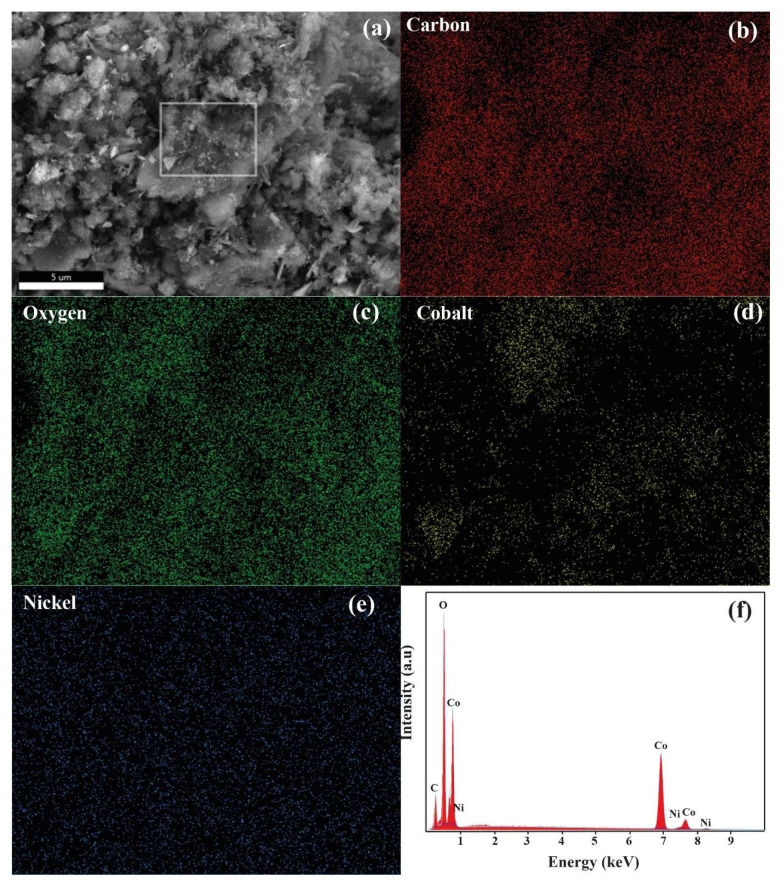
SEM image (a), elemental mapping of carbon (b), oxygen (c), cobalt (d), nickel (e), and EDX spectrum of NiNPs@Co_3_O_4_/GO-COOH (f).

The surface properties of the materials obtained were assessed by means of SEM. Figure 1a shows a characteristic SEM image of the prepared GO-COOH with a 2-μm scale bar [33]. Figure 1b displays the surface of the Co_3_O_4_ nanosheets with 2-μm scale bar. The general morphology of the synthesized Co_3_O_4_ nanosheets is displayed in Figure 1b and they exhibit sheet-like shapes less than 6 μm in length on average. Furthermore, the nanosheets’ surface appears to be extremely coarse and exhibits a comparatively porous surface shape. The porous nanoplates were made up of numerous equable nanoparticles with a mean diameter of about 95 nm, as the magnified SEM image made evident. As shown in Figure 1c, the estimated thickness of the nanoplates was about 120 nm. The SEM image of Co_3_O_4_/GO-GOOH is shown in Figure 1c. Co_3_O_4_ nanoplates were dispersed in the GO-COOH layers, as the image illustrates. Figure 1d depicts the SEM image of NiNPs@Co_3_O_4_/GO-COOH composite. Then the elemental mapping and EDX spectrum of the composite was obtained, as illustrated in Figure 2.

**Figure 3:**
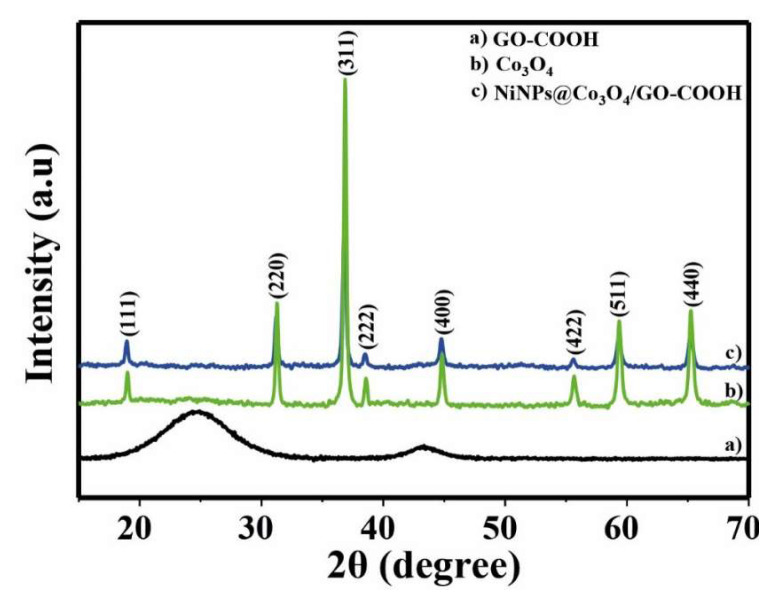
XRD graphs of GO-COOH (a), Co_3_O_4_ (b), and NiNPs@Co_3_O_4_/GO-COOH (c).

XRD was carried out to assess the crystallography of the materials produced. The resulting XRD patterns for the materials are shown in Figure 3. As illustrated in Figure 3a, the excellently described 2θ peaks at 19.01°, 31.27°, 36.87°, 38.60°, 44.82°, 55.67°, 59.37°, and 65.25° are ascribed to the formation of Co_3_O_4_ (JCPDS 42-1467) [34]. The unwanted phases of Ni clusters or NiO-based peaks are not seen in the XRD patterns. Additionally, once Ni was deposited on the surface of Co_3_O_4_/GO-COOH, the peak at 44.82° seems to move slightly to the left (44.78°), suggesting the existence of Ni nanoparticles [35]. The 2θ peaks observed at 24.46° and 43.34° are responsible for the characteristic peak of GO-COOH [36].

**Figure 4:**
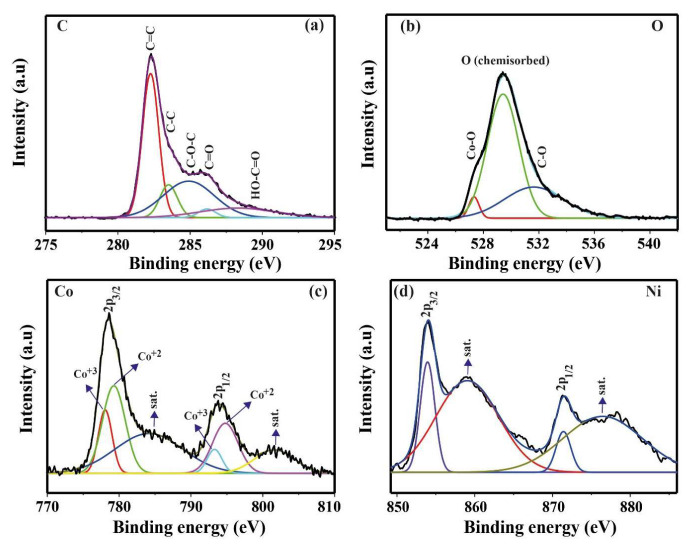
XPS high-resolution spectra of C1s (b), O1s (c), Co2p (c), and Ni2p (d).

The chemical structure of the NiNPs@Co_3_O_4_/GO-COOH composite was better understood by using XPS. As shown in Figure 4a, the XPS peaks achieved at 282.27, 283.43, 284.93, 286.2, and 288.8 eV are attributed to C=C (sp^2^), C–C (sp^3^), C–O–C, C=O, and COOH or HO–C=O groups in GO-COOH, respectively [37]. To accurately assess the reactivity and activation process of surface lattice oxygen, the XPS data of O 1s were obtained and then fitted into various oxygen species. As can be seen in Figure 4b, the observed peaks at 527.36, 529.42, and 531.61 are ascribed to Co–O, chemisorbed of oxygen, and C–O groups, respectively [38]. Figure 4c displays the XPS spectra of Co2p. The two peaks observed at 778.54 eV and 793.85 eV are associated with spin orbit peaks of 2p_3/2_ and 2p_1/2_, respectively, which indicates that the oxidation case of Co^2+^ and the spin orbit splitting energy value is 15.31 eV in NiNPs@Co_3_O_4_/GO-COOH. The peaks observed at 778.04/793.27 eV and 779.26/794.80 eV are associated with Co^3+^ and Co^2+^, respectively. Moreover, the peaks obtained at 784.17 eV and 801.60 eV are ascribed to the satellite peaks [39]. Figure 4d displays the XPS spectrum of Ni2p. Ni2p3/2 and Ni2p1/2 are represented by the two primary peaks in the spectrum, which are located at 853.92 eV and 871.42 eV, respectively; the spectrum peak separation of Ni2p was 17.46 eV. Additionally, the peaks observed at 859.05 eV and 876.41 eV are associated with the satellite peaks of Ni^2+^ and Ni^3+^ [40].

### 3.2. Electrochemical studies

#### 3.2.1. Cyclic voltammetry (CV) experiments

**Figure 5:**
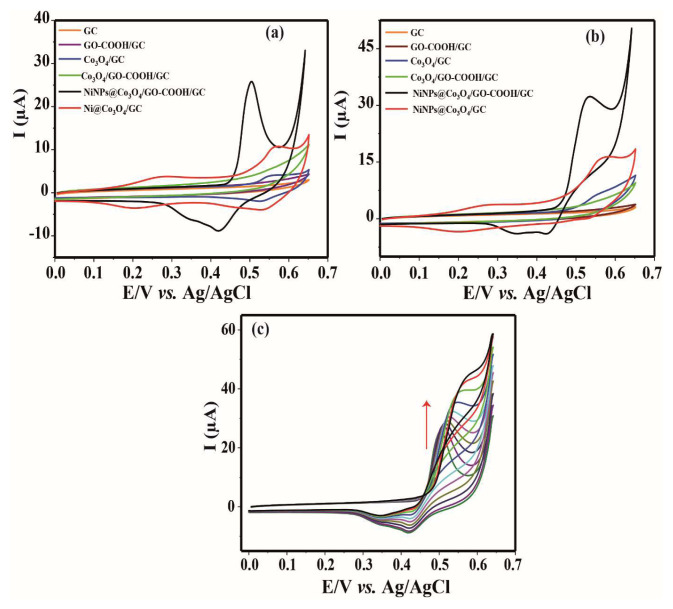
Cyclic voltammograms obtained on the working electrodes in 0.1 M NaOH solution in the absence of glucose (a), in the presence of 0.6 mM glucose at 0.05 V s^−1^ (b), and cyclic voltammograms achieved on the NiNPs@Co_3_O_4_/GO-COOH/GC electrode in 0.1 M NaOH solution containing various glucose concentrations at 0.05 V s^−1^ (c).

The electrocatalytic performance of both bare GC and modified GC electrodes was investigated using CV in 0.1 M NaOH at the scanning rate of 50 mV s^−1^. The influence of various components such as Co_3_O_4_ and NiNPs@Co_3_O_4_/GO-COOH on the current response to glucose was evaluated in the absence (Figure 5a) and in the presence of glucose (Figure 5b). In the CV results without the target analyte, at the bare GC electrode, an almost flat line was achieved, showing no significant signal response. In addition, no obvious oxidation peak was observed on the GO-COOH/GC electrode. However, on the Co_3_O_4_-modified GC electrode (a), relatively small cathodic and anodic peaks were displayed at 0.527 V and 0.552 V, respectively. Similarly, the NiNPs@Co_3_O_4_/GO-COOH/GC electrode showed two reduction and one oxidation peaks that were clear and well-defined with the highest current response at 0.349 V, 0.420 V, and 0.505 V, respectively. The oxidation and reduction peak currents are ascribed to the formation of the Ni^2+^/Ni^3+^ and Co^3+^/Co^4+^ redox couple. The absorbed water molecules on the NiNPs@Co_3_O_4_/GO-COOH/GC electrode surface cause the nickel nanoparticles to convert into Ni(OH)_2_ and eventually NiOOH, and Co_3_O_4_ to CoO_2_ and finally CoOOH [41]. Figure 5b depicts the cyclic voltammograms at various electrodes (GC, GO-COOH/GC, Co_3_O_4_/GC, Co_3_O_4_/GO-COOH/GC, NiNPs@Co_3_O_4_/GC, and NiNPs@Co_3_O_4_/GO-COOH/GC electrodes) in 0.1 M NaOH containing 0.6 mM glucose. The NiNPs@Co_3_O_4_/GO-COOH/GC modified electrode had the highest current intensity of the redox peaks among the electrodes assessed, with well-described anodic and cathodic peak current values. The anodic peak current was observed at 0.535 V and the anodic peak currents were seen at 0.425 V and 0.349 V for 0.6 mM glucose, demonstrating that the NiNPs@Co_3_O_4_/GO-COOH/GC electrode showed notable electrocatalytic activity for the oxidation of glucose. The following mechanism can be proposed for the reaction in the alkaline environment:


(1) $$\text{Ni}+{\text{Co}}_{3}{\text{O}}_{4}+2{\text{OH}}^{-}+{\text{H}}_{2}\text{O}↔\text{Ni}{\left(\text{OH}\right)}_{2}+3\text{CoOOH}+3{\text{e}}^{-}$$

(2) $$\text{Ni}{\left(\text{OH}\right)}_{2}+{\text{OH}}^{-}\to \text{NiOOH}+{\text{H}}_{2}\text{O}+{\text{e}}^{-}$$

(3) $$\text{CoOOH}+{\text{OH}}^{-}↔{\text{CoO}}_{2}+{\text{H}}_{2}\text{O}+{\text{e}}^{-}$$

(4) $$2{\text{CoO}}_{2}+\text{Glucose}\to 2\text{CoOOH}+\text{Gluconolactone}$$

(5) $$\text{NiOOH}+\text{Glucose}\to \text{Ni}{\left(\text{OH}\right)}_{2}+\text{Gluconolactone}$$

The electrochemical oxidation of glucose to gluconolactone catalyzed by the NiNPs@Co_3_O_4_/GO-COOH/GC working electrode caused an enhancement of oxidation current. Furthermore, the unique structure of GO-COOH and the synergistic impact between NiNPs and Co_3_O_4_ were crucial in raising the oxidation peak current value for the determination of glucose. Figure 5c displays the cyclic voltammograms achieved on the NiNPs@Co_3_O_4_/GO-COOH/GC electrode in 0.1 M NaOH solution including various concentrations of glucose at the scan rate of 0.05 V s^−1^. As demonstrated in the figure, the oxidation peak current associated with the electrochemical oxidation of glucose rises in direct proportion to the amount of glucose.

**Figure 6:**
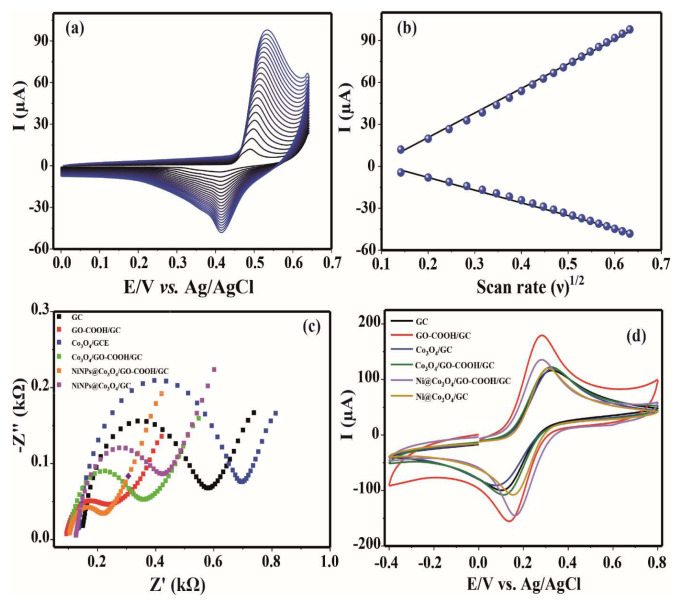
Cyclic voltammograms obtained on the NiNPs@Co_3_O_4_/GO-COOH/GC electrode 0.1 M NaOH solution containing 0.2 mM glucose using various scan rates (from 20 to 400 mV s^−1^) (a), current vs. square root of scan rate graph (b), Nyquist graph achieved under the applied potential of 0.2 V, the frequency range of 100 kHz to 0.1 Hz, and amplitude of 0.01 V (c), cyclic voltammograms (d) of the working electrodes taken in 5.0 mM [Fe(CN)_6_]^3−^/[Fe(CN)_6_]^4−^ including 0.1 M KCl at the scan rate of 0.1 V s^−1^.

In order to comment on the impact of the potential scan rate on the NiNPs@Co_3_O_4_/GO-COOH/GC electrode, cyclic voltammograms were carried out in 0.1 M NaOH containing 0.2 mM glucose at various scan rates. As can be seen in Figure 6a, with a scan rate between 20 and 400 mV s^−1^, the oxidation peak potential moved to more positive potentials, indicating the kinetic limits of the electrochemical process [42]. In addition, the oxidation peak current increased linearly with increasing square root of the scan rate (Figure 6b), which demonstrates a diffusion-controlled electrochemical process [43] .

Figure 6c exhibits a typical Nyquist graph comprising a semicircle part seen at higher frequencies and a linear part observed at lower frequencies. The diameter of the semicircle part equals the charge transfer resistance (Rct), which is attributed to the electron-transfer kinetics of the redox couple ([Fe(CN)_6_]^3−^/[Fe(CN)_6_]^4−^) at the working electrode surface, and the linear part presents the diffusion limited process [44]. The Rct value of the working electrode was 420 Ω for the bare GC electrode, 171 Ω for the GO-COOH/GC electrode, 569 Ω for the Co_3_O_4_/GC electrode, 301 Ω for the NiNPs@Co_3_O_4_/GC electrode, 234 Ω for the Co_3_O_4_/GO-COOH/GC electrode, and 131 Ω for the NiNPs/Co_3_O_4_/GO-COOH/GC electrode, which demonstrates that the NiNPs increase the conductivity or decrease the charge transfer resistance of the working electrode. Figure 6d shows the cyclic voltammograms obtained in 5 mM Fe(CN)_6_]^3−^/[Fe(CN)_6_]^4−^ (1:1) solution containing 0.1 M KCl at the scan rate of 0.1 V s^−1^. The oxidation peak current value was estimated as 104.73 μA for the GC electrode, 150.21 μA for the GO-COOH/GC electrode, 107.84 μA for the Co_3_O_4_/GC electrode, 112.63 μA for the NiNPs@Co_3_O_4_/GC electrode, 109.13 μA for the Co_3_O_4_/GO-COOH/GC electrode, and 118.93 μA for the NiNPs/Co_3_O_4_/GO-COOH/GC electrode. The anodic peak current of the probe on the GO-COOH/GC electrode was higher than that on the NiNPs/Co_3_O_4_/GO-COOH/GC electrode. The use of hydrazine hydrate in nanocomposite synthesis reduced the oxidation peak current because it partially removed oxygen-containing functional groups from the composite. This is because, according to previous studies, the functional groups containing oxygen increase the oxidation peak current [26,45].

The Randles–Sevcik equation was performed to detect the active surface area of the bare and modified working electrodes [46]:


(6) $$I_{p}=(2.69\times 10^{5})n^{3/2}D_{0}^{1/2}C_{0}A\nu^{1/2}$$

**Figure 7:**
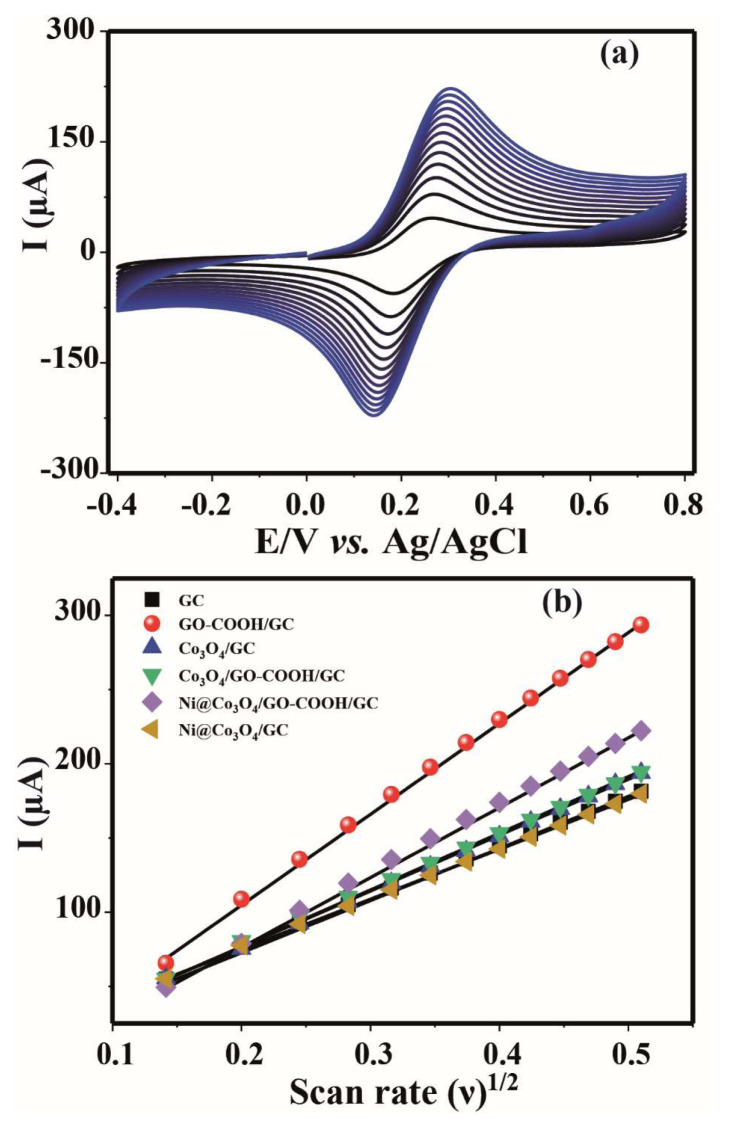
Cyclic voltammograms (a) and current versus square root of scan rate graph of the NiNPs@Co_3_O_4_/GO-COOH/GC electrode obtained in 5.0 mM [Fe(CN)_6_]^3−^/[Fe(CN)_6_]^4−^ including 0.1 M KCl at different scan rates (20–260 mV s^−1^).

In the above equation, *I_P_* is the anodic peak current in amperes (A), n is the electrode number taking part in the electrochemical reaction, *D*_0_ is the diffusion coefficient in cm^2^ s^−1^, *C*_0_ is the concentration of the probe (5 mM [Fe(CN)_6_]^3−^/[Fe(CN)_6_]^4−^) in mol cm^−3^, and *v* is the scan rate in V s^−1^. The active surface area was 0.177 cm^2^ for the GO-COOH/GC electrode, 0.108 cm^2^ for the Co_3_O_4_/GC electrode, 0.103 cm^2^ for the NiNPs@Co_3_O_4_/GC electrode, 0.110 cm^2^ for the Co_3_O_4_/GO-COOH/GC electrode, and 0.136 cm^2^ for the NiNPs@Co_3_O_4_/GO-COOH/GC electrode (Figures 7a and 7b). According to these results, the active surface area of the working electrode increased by approximately 23.64% after Ni particles were deposited on the Co_3_O_4_/GO-COOH composite.

#### 3.2.2. Amperometric experiments

**Figure 8:**
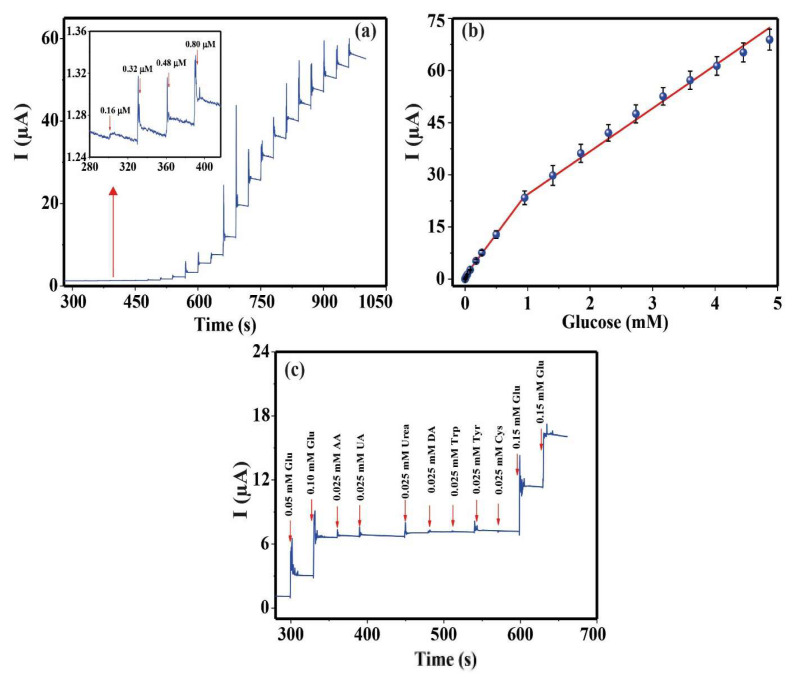
Amperometric graph of the NiNPs@Co_3_O_4_/GO-COOH/GC electrode obtained in 0.1 M NaOH at 0.55 V (b), current vs. glucose (mM) graph, (c) amperometric response of the NiNPs@Co_3_O_4_/GO-COOH/GC electrode to glucose, and some electroactive compounds achieved in 0.1 M NaOH at 0.55 V.

The anodic peak current was measured using various glucose concentrations to assess the sensing performance of the NiNPs/Co_3_O_4_/GO-COOH/GC electrode. The amperometric response of the fabricated sensor to the target analyte is based on the potential applied. Thus, the potential applied for the electrooxidation of glucose was selected as +0.55 V. Figure 8a shows the typical amperometric i–t graph observed at the NiNPs/Co_3_O_4_/GO-COOH/GC electrode in 0.1 M NaOH solution by addition of different glucose concentrations at +0.55 V. The electrochemical signal response became stable in a few seconds (1–5 s) as stated in previous similar articles [47,48]. As displayed in Figure 8b, the sensor exhibited two dynamic linear detection ranges. The first one was 4.8 × 10^−7^–9.5 × 10^−4^ M and the second one was 9.5 × 10^−4^–4.87 × 10^−3^ M. The linear equations were (μ*A*) =24.747 × [*Glucose*](*mM*) + 0.2614 (R^2^ = 0.9973) and I (μ*A*) =11.612 × [*Glucose*](*mM*) + 14.338 (R^2^ = 0.9923). The LOD was estimated to be 0.14 μM using a signal-to-noise ratio of 3. The sensitivities were computed to be 181.96 μA mM cm^−2^ and 85.38 μA mM cm^−2^ depending on the active surface area of the sensor. The regression value of the calibration lines was detected and is displayed in Table 1. Additionally, the electrocatalytic response of the NiNPs/Co_3_O_4_/GO-COOH/GC electrode was compared with that previously reported (Table 2). For example, in one study, glucose was determined nonenzymatically using a Ni-NPs/TiO_2_-NTs-based electrochemical sensor. The sensor showed a linear detection range of 0.004–4.8 mM for glucose. The sensor also exhibited a LOD of 2 μM and a sensitivity of 700.2 μA mM cm^−2^ [49]. In a study by Ensafi and coworkers, a Ni@PS-CPE-based nonenzymatic glucose sensor was developed. The sensor showed a detection range of 2–5000 μM for glucose and the LOD calculated was 0.2 μM [50]. In another work, nanorod-like Ni nanoparticles deposited on porous carbon nanorods were synthesized. The nanocomposite-modified GC electrode was used for nonenzymatic electrochemical glucose detection. The sensor exhibited two linear detection ranges (0.0001–0.5336 mM with sensitivity of 337.32 μA cm^−2^ mM^−1^ and 0.5336–3.03 mM with sensitivity of 210.56 μA cm^−2^ mM^−1^). The LOD estimated was 0.07 μM [51]. The linear detection range, LOD, and sensitivity obtained in the present study, when compared with previous studies, show that the NiNPs/Co_3_O_4_/GO-COOH/GC sensor provides satisfactory results.

#### 3.2.3. Selectivity, reusability, reproducibility, and real sample experiments

One of the most important performance factors indicating the usability of an electrochemical sensor/biosensor is undoubtedly its selectivity. In our study, highly electroactive compounds such as UA, AA, and DA were used to evaluate the selectivity of the NiNPs/Co_3_O_4_/GO-COOH/GC electrode. Considering the glucose content in biological material such as serum, 0.025 mM of each electroactive compound was used against glucose concentrations of 0.05, 0.10, and 0.15 mM. Figure 8c shows a typical amperometric graph, i.e. a current–time graph. The response value was +4.44% for AA, +3.31% for UA, +7.80% for urea, 1.49% for DA, 0.38% for Trp, 3.45% for Tyr, and −0.35% for Cys. Considering the level of glucose in the serum, the sensor showed satisfactory selectivity for glucose among the compounds likely to be found in biological fluids.

In electrochemical sensors, repeatability is a performance factor that indicates how consistently a sensor can produce repeatable results in successive measurements under the same conditions (such as the same analyte concentration, operating temperature, and preparation procedure). The sensor’s ability to produce highly repeatable results increases its reliability. Therefore, in our study, to investigate the repeatability of the NiNPs/Co_3_O_4_/GO-COOH/GC electrode, seven different glucose concentrations (2, 4, 10, 20, 50, 100, and 250 μM) were used and four measurements were obtained for each of these concentrations using the same sensor. Relative standard deviation (RSD) was estimated to be 7% for 2 μM, 4.08% for 4 μM, 4.47% for 10 μM, 7.22% for 20 μM, 4.32% for 50 μM, 4.16% for 100 μM, and 2.38% for 250 μM, demonstrating satisfactory reusability for the sensor. The reproducibility of the sensor was investigated using different electrodes prepared under the same conditions and various glucose concentrations. RSD for reproducibility was 5.13% for 2 μM, 3.59% for 4 μM, 4.94% for 10 μM, 4.85% for 20 μM, 6.07% for 50 μM, 5.05% for 100 μM, and 2.78% for 250 μM, demonstrating satisfactory reusability for the sensor. Furthermore, the storage stability of the sensor was tested for 20 days. After 20 days, the sensor was still found to be responding at (96 ± 3.05)% of its initial activity level, demonstrating good storage stability.

The NiNPs/Co_3_O_4_/GO-COOH-modified GC electrode was used to detect glucose in human serum and spiked glucose solutions in order to evaluate its applicability in real samples. Amperometry was used to quantitate glucose in the samples. The estimated glucose concentrations in the serum, which agreed with the glucose meter’s readings, are seen in Table 3. Student’s t-test with a 97.5% confidence level was used in the statistical computations for the amount of glucose. The recovery was estimated to be between 97.64% and 100.69%, which demonstrate that the constructed NiNPs/Co_3_O_4_/GO-COOH/GC electrode can be used to determine glucose in real samples.

## 4. Conclusion

We present a novel enzyme-free electrochemical glucose sensor depending upon a NiNPs/Co_3_O_4_/GO-COOH nanocomposite-modified GC electrode. The sensor showed two linear determination ranges for glucose. The first linear range was 4.8 × 10^−7^–9.5 × 10^−4^ M with sensitivity of 181.96 μA mM cm^−2^ and the second linear range was 9.5 × 10^−4^–4.87 × 10^−3^ M with sensitivity of 85.38 μA mM cm^−2^. The LOD was 0.14 μM. The sensor showed good selectivity for glucose compared to electroactive compounds such as AA, DA, and UA. Seven different glucose concentrations were studied to assess the repeatability and reproducibility of the sensor. For repeatability, RSD values were in the range of 2.38% to 7.22%. For reproducibility, RSD values were in the range of 2.78% to 6.07%, indicating that the sensor had good repeatability and reproducibility. These results indicate that the sensor has satisfactory performance factors and could be a good reference for future sensor research.

## Figures and Tables

**Table 1:** The regression value of the calibration lines for the electrochemical determination of glucose using the NiNPs@Co_3_O_4_/GO-COOH/GC sensor.

Linear detection range	0.00048–0.95 mM 0.95–4.87 mM
Regression equation with R^2^	y=24.747x+0.2614 (0.9973) y=11.612x+14.338 (0.9923)
Sensitivity	181.96 μA mM^−1^ cm^−2^ 85.35 μA mM^−1^ cm^−2^
LOD	0.14 μM
LOQ	0.47 μM
Standard error	0.3603 0.3609

**Table 2:** The comparison of the performance factors of some nonenzymatic glucose sensors.

Electrode	Linear range (mM)	Sensitivity (μA mM^−1^ cm^−2^)	LOD (μM)	Ref.
NC-MOFs@CCFs	0.04–3.13 3.63–8.28	105.2 23	0.116	[8]
BDND nanoflowers	0–6	2.1952	9.0	[16]
a-Ni_2_/Co_1_-NC/CC	0.002–2 2–6	2455 1300	0.15	[24]
NiO-Co_3_O_4_/NF/Pt	0.0005–6.5	1934.2	0.15	[41]
NiVP/Pi	0.0001–0.001 0.001–10	1.130 0.746	0.0037	[44]
Cu/graphene	up to 4.5	-	0.5	[48]
Ni-NPs/TiO_2_NTs	0.004–4.8	700.2	2	[49]
Ni@PSi	0.002–5	-	0.2	[50]
Ni/NCNs-500	0.0001–0.5336 0.5336–3.03	337.32 210.56	0.07	[51]
NiNPs@Co_3_O_4_/GO-COOH/GC	0.00048–0.95 0.95–4.87	181.96 85.38	0.14	This work

**Table 3:** Detection of glucose in serum and spiked samples using the NiNPs@Co_3_O_4_/GO-COOH/GC sensor.

Samples	Added (mg/dL)	Glucose meter (mg/dL)	NiNPs@Co_3_O_4_/GO-COOH/GC (mg/dL)	RSD%	Recovery%
Serum	-	83	81.04 ± 3.04	3.02	97.64
Glucose	18	-	17.59 ± 0.71	3.14	97.72
Glucose	90	-	88.81 ± 6.98	6.25	98.68
Glucose	180	-	181.24 ± 8.75	3.92	100.69
Glucose	360	-	360.60 ± 14.06	3.15	100.17
